# The HGF inhibitory peptide HGP-1 displays promising *in vitro* and *in vivo* efficacy for targeted cancer therapy

**DOI:** 10.18632/oncotarget.3937

**Published:** 2015-05-11

**Authors:** Lisha Chen, Chunlin Li, Yimin Zhu

**Affiliations:** ^1^ Key Laboratory of Nano-Bio Interface, Division of Nanobiomedicine, Suzhou Institute of Nano-Tech and Nano-Bionics, Chinese Academy of Sciences, Suzhou 215123, China; ^2^ Suzhou Institute of Nano-Tech and Nano-Bionics, CAS, University of Chinese Academy of Sciences, Beijing 100049, China

**Keywords:** HGF targeting peptide, HGF/MET signaling pathway, cancer targeted therapy

## Abstract

HGF/MET pathway mediates cancer initiation and development. Thus, inhibition on HGF-initiated MET signaling pathway would provide a new approach to cancer targeted therapeutics. In our study, we identified a targeting peptide candidate binding to HGF which was named HGF binding peptide-1 (HGP-1) via bacterial surface display methods coupled with fluorescence-activated cell sorting (FACS). HGP-1 showed the moderate affinity when determined with surface plasmon resonance (SPR) technique and high specificity in binding to HGF while assessed by fluorescence-based ELISA assay. The results from MTT and *in vitro* migration assay indicated that HGF-dependent cell proliferation and migration could be inhibited by HGP-1. *In vivo* administration of HGP-1 led to an effective inhibitory effect on tumor growth in A549 tumor xenograft models. Moreover, findings from Western Blots revealed that HGP-1 could down-regulated the phosphorylation levels of MET and ERK1/2 initiated by HGF, which suggested that HGP-1 could disrupt the activation of HGF/MET signaling to influence the cell activity. All the data highlighted the potential of HGP-1 to be a potent inhibitor for HGF/MET signaling.

## INTRODUCTION

The c-mesenchymal-epithelia transform factor (c-MET, also called MET) was first discovered in the 1980s as an activated oncogene [1], and hepatocyte growth factor (HGF) was the sole physiological ligand of MET [2]. The binding activity of HGF to MET activates downstream signaling pathway, which regulates cell mitogenesis, motogenesis, angiogenesis and morphogenesis [3, 4]. The dysregulation of HGF/MET pathway has been implicated in many kinds of cancers [5–7]. MET is frequently involved in the pathogenesis of solid tumors. For example, MET receptor overexpression is often observed in gastric carcinoma, medulloblastoma, glioblastoma and non-small cell lung cancer [1, 8, 9]. MET mutation is another MET-related dysregulation that exists in colorectal cancer [1]. Meanwhile, the disorders of HGF also play important roles in tumors progression. HGF autocrine and overexpression are inducers of angiogenesis in brain cancer [1, 8, 10]. The mutation of HGF in breast cancer is related to its progression [11]. Moreover, the expression level of HGF in the tumor tissue is a crucial index for prognosis evaluation in some cases such as glioma, due to its higher concentration in malignant glioma than adenomas, oligodendrogliomas and normal brain tissues [12]. Therefore, development of agents targeting to components in HGF/MET pathway is a potential therapeutic strategy for many cancers.

In recent years, many HGF/MET-related targeting inhibitors or competitors are on the way to pre-clinical or clinical evaluation. Various HGF targeting agents including HGF antibodies and bio-antagonists are reported. NK4 [13], a well-known HGF antagonist, is a potent competitor that disrupts HGF binding to MET. Ficlatuzumab [8, 14, 15] is one of HGF antibodies undergoing clinical evaluation. Antibodies targeting MET, including single-armed humanized monovalent antibodies such as onartuzumab, are under phase II clinical evaluation [1]. A novel targeting protein, anticalin PRS-110 is an effective MET targeting agents [16]. In addition, small-molecular tyrosine kinase inhibitors (TKIs) are other important parts of MET targeting agents. Some of TKIs have been evaluated in pre-clinical models. Among them, PHA665752 and SU11274 from Pfizer are the typical representatives [1, 8].

Although the existing MET or HGF targeting agents including TKIs and antibodies exhibit effective inhibition on MET signaling [17], they have some deficiencies in clinical application. For instance, antibodies with large molecular weight will lead to relatively low tissue penetrability. In addition, antibodies have the potential to cause receptor dimerization without ligands due to their bivalent structure of immunoglobulins [8], which may create potential risk in clinical targeting therapeutics. As for TKIs, their broaden spectrum of inhibition on tyrosine kinases may result in serious side effects [16]. Furthermore, the acquired resistance in MET targeted therapy initiated by TKIs [18] would limit their application in targeting therapy as well.

Targeting peptides, biomolecules with lower molecular weight and good tissue penetration, emerge as an attractive class of therapeutic agents. In 1992, there existed reports on peptides application in lymphoma therapy [19]. Benefiting from bacterial surface display, we obtained a series of HGF targeting peptides. By assessing the *in vitro* physicochemical activities and *in vivo* bioactivities, a HGF targeting peptide was selected to be a potential inhibitor candidate for HGF/MET signaling pathway.

## RESULTS

### Identification of binding peptides for HGF from a fully random bacteria display library

**Figure 1:**
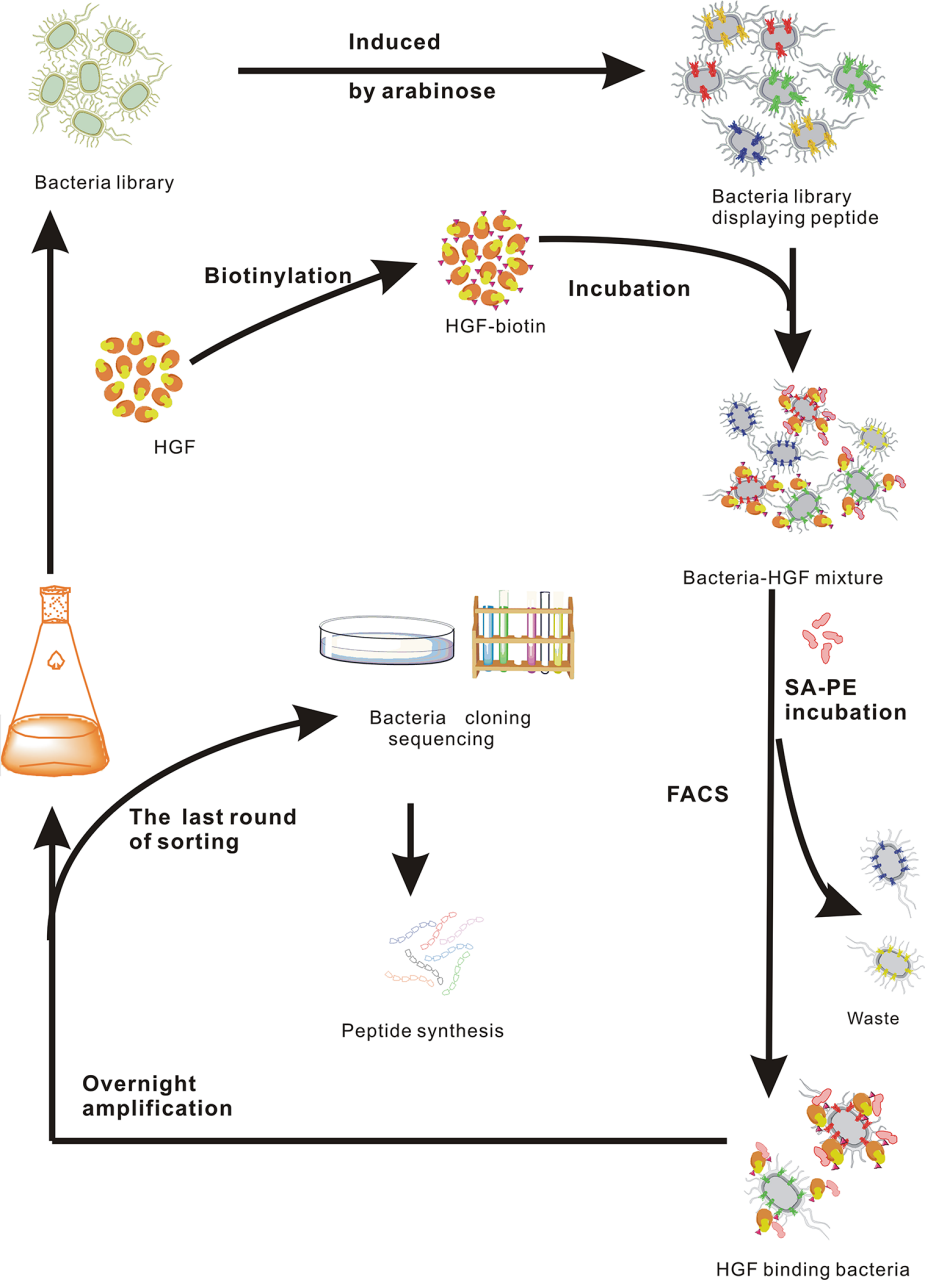
Schematic illustration of HGF targeting peptide screening by FACS

**Figure 2:**
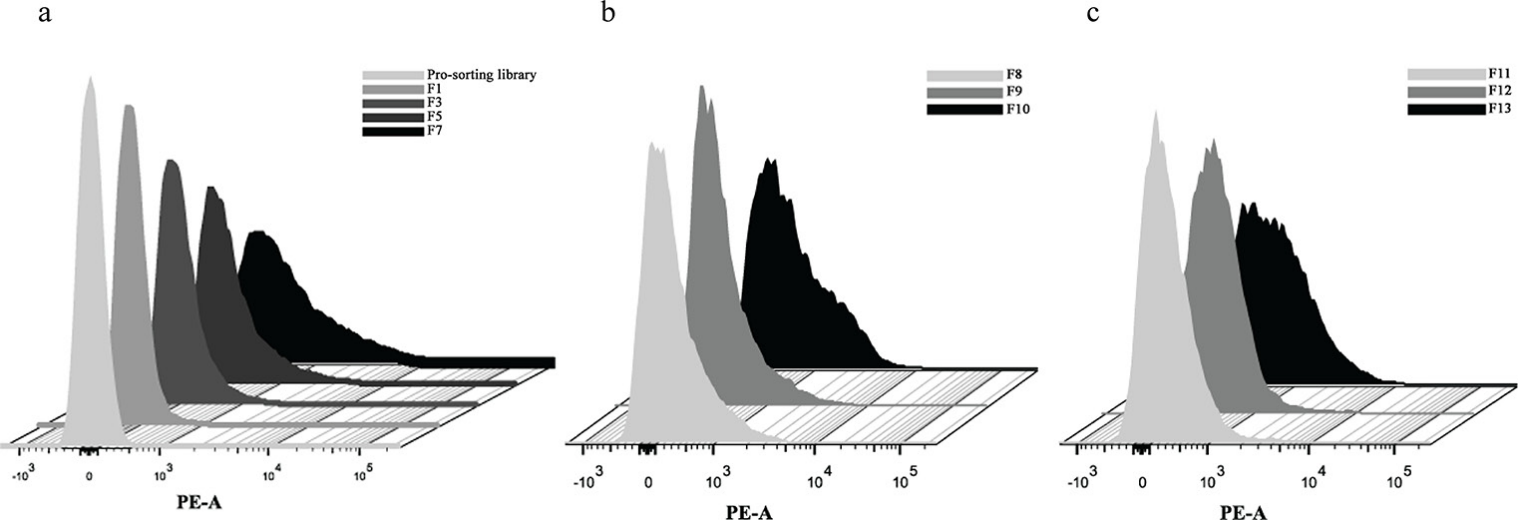
HGF binding peptides were enriched by bacteria surface display coupled with FACS **a.** Fluorescence intensity in sorting cycle 1–7 (21 nM HGF). **b.** Fluorescence intensity in sorting cycle 8–10 (10% human serum and 10 nM HGF). **c.** Fluorescence intensity in sorting cycle 11–13 (10% human serum and 5 nM HGF).

To identify the peptide sequences binding to HGF, a fully random 15-mer bacteria peptide library (X15) was used. A schematic illustration for fluorescence-activated cell sorting (FACS) was shown (Figure 1). In order to screen the HGF binding peptides effectively and reduce the library size rapidly, the original library was sorted by one cycle of magnetic cell sorting (MACS). Through MACS and 7 cycles of FACS, percentages of bacteria in the sorting gate increased from 2.3% to 50.5% (Supplementary Figure S1), and PE-A fluorescence intensity of whole population in each cycle ascended from 33 to 851 (Figure 2a). In addition, to obtain peptides with higher affinity and specificity to HGF, the incubation concentration of HGF was decreased coupling with adding 10% human serum into the mixture in the following generations of sorting. After next 6 cycles of screening, there was a significant increase in the mean intensity of PE-A fluorescence of enriched libraries (Figure 2b and 2c). Totally 52 bacteria clones were selected for sequencing and 18 different peptide sequences were obtained (Table 1). No obvious consensus sequence was identified.

**Table 1:** The sequences of the HGF binding peptides

Clone	Peptide sequence	Frequency	Percentage
1	HRGLKWEIVPWSGCG	14	28.6%
2	TLYEVDLREWCAGIVG	7	14.3%
3	TYFTWWELSPGCEEH	6	12.2%
4	RANWFCIEDSVYCGK	3	6.1%
5	SARKLGWCPYWSSDC	3	6.1%
6	MEVERRWPWWHANYW	3	6.1%
7	AYMMRDLWEYNWTSG	2	4.1%
8	KGYGRYWWDCGDNFW	1	2.0%
9	YWPGCQEWGNKWWGG	1	2.0%
10	YRWGMGGYEYWCNRG	1	2.0%
11	QRCGMVRFNDWQHPM	1	2.0%
12	GTARLLWRPVVTYDC	1	2.0%
13	MGGGSWLYVGDPDCW	1	2.0%
14	RSSMWDCYYFDCCNW	1	2.0%
15	ALQQAWGWWDCYGWR	1	2.0%
16	MYNVRLNDWYWCGWD	1	2.0%
17	SPWWSVYETQSCAVR	1	2.0%
18	YWYGWGSGWSGCDHA	1	2.0%

### The binding capability and specificity of peptides displayed on the surface of bacteria

The peptides displayed on the surface of bacteria clone 1, 2 and 3 were named HGF binding peptide-1, 2 and 3 (HGP-1, 2 and 3) separately. Peptide physicochemical properties were preliminarily analyzed with bacteria clones. To evaluate the binding ability of peptides on the surface of bacteria to HGF, the PE-A fluorescence intensity of each clone with different wash procedures were determined by flow cytometry. Bacteria clones undergoing regular or stringent washing procedures after incubation with HGF were prepared. Results from flow cytometry showed that HGP-1 and HGP-3 in washed groups bound to their targets as well as the regular ones, but HGP-2 in washed group had an obvious reduction (Supplementary Figure S2a). These data indicated that HGP-1 and HGP-3 had higher binding capability to HGF compared with HGP-2 when displayed on the surface of bacteria.

To evaluate binding specificity of peptide displayed on the surface of bacteria, cytokines including VEGF, EGF, bFGF and BSA were added separately into the incubation system. All samples were prepared referring to procedure in sorting and their PE-A fluorescence intensities were measured by flow cytometry. These proteins or cytokines, except for EGF, did not disrupt bacteria clones binding with HGF (Supplementary Figure S2b).

### The binding properties of soluble peptides to HGF

In order to gain an insight into the binding capability of bacteria-free HGF binding peptides, the peptides were synthesized and their binding properties were determined. Moreover, a scrambled peptide of HGP-1 (SP-H1) was designed as described previously [20, 21]. To verify that SP-H1 could not bind to HGF, increasing concentrations of SP-H1 were incubated with HGF coated on the plate. Results from the fluorescence-based ELISA assay demonstrated that SP-H1 remained a low binding level to HGF at a high concentration (100 μM) (Supplementary Figure S3a). It indicated that SP-H1 could be an appropriate control in the study.

**Figure 3:**
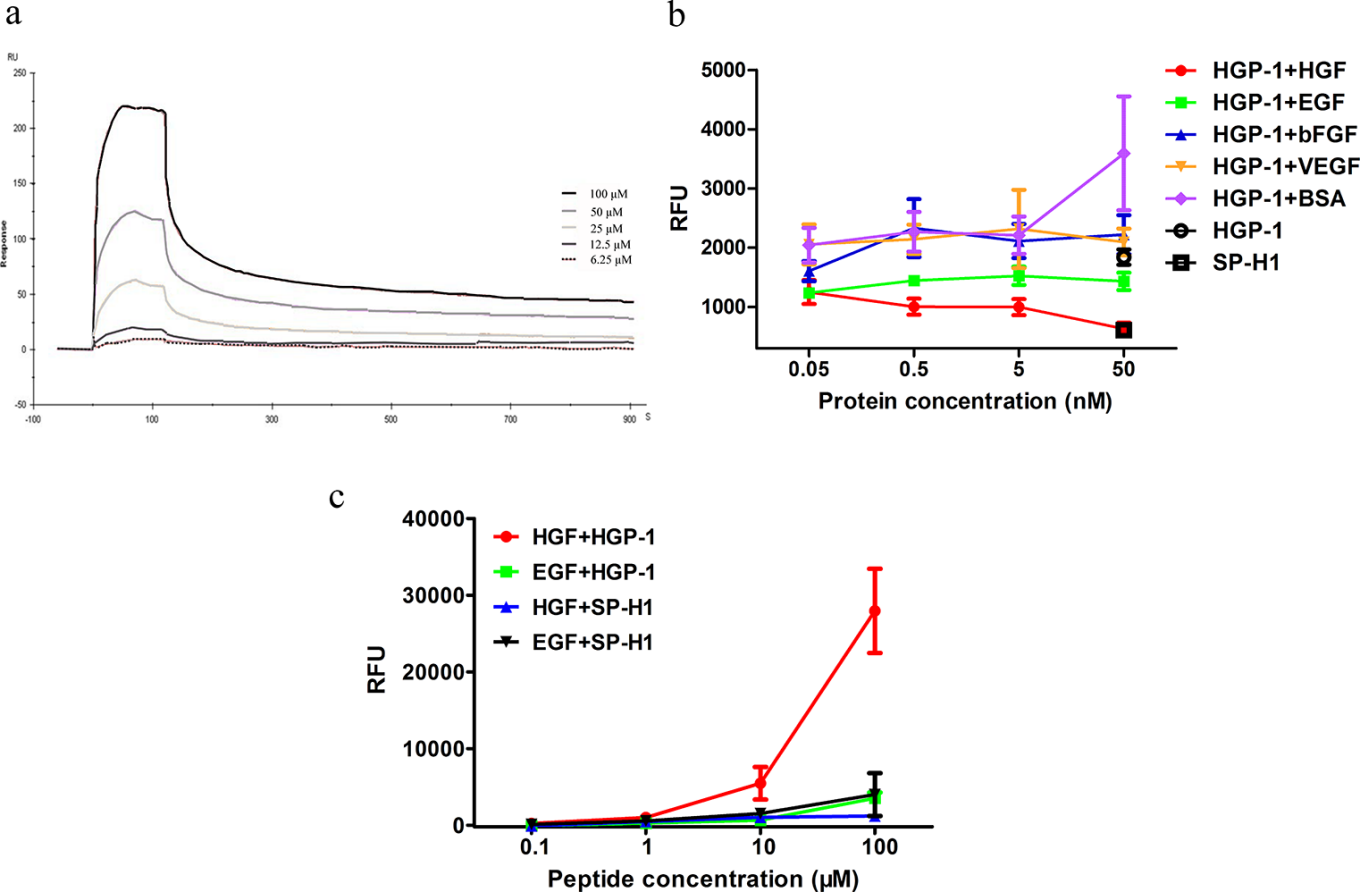
Physicochemical properties of HGF targeting peptide **a.** *K_D_* of HGP-1 binding to HGF was determined by SPR technique. **b.** The assessment of binding competition between various proteins and HGF by fluorescence-based ELISA assay post 1.5-hour incubation. Proteins at the concentrations of 0.05 nM, 0.5 nM, 5 nM and 50 nM mixed with 10 μM FITC-labeled HGP-1 were the liquid phase (*n* = 5). **c.** The binding activity between HGP-1 to HGF and EGF were measured by fluorescence-based direct ELISA assay post 1.5-hour incubation. HGP-1 at the concentrations of 0.1 μM, 1 μM, 10 μM, 100 μM were used (*n* = 3). Values were mean ± SEM.

Since HGP-1 and HGP-2 showed different binding capability on the surface of bacteria, we decided to examine the binding properties of these two peptides. To pre-assess the binding ability of peptide to HGF, peptides were coated on the fluorescence plates and incubated with HGF. The well coated with HGP-1 had at least 1500 times higher RFU than the one with SP-H1. HGP-2 had a weaker binding with HGF, but still with 700 times much higher RFU than SP-H1 when coated on the well (Supplementary Figure S3b). In other words, HGP-1 performed a twice much stronger binding capability to HGF than HGP-2, which was in accordance with results from relative bacteria clones. In order to obtain a precise affinity data of HGP-1, the affinity and kinetics of HGP-1 against HGF was detected by SPR technique (Figure 3a). HGF was immobilized on the surface of a CM5 chip. Then, the active groups were inactive by ethanolamine. Different concentrations of HGP-1 were injected over the surface of the chip followed by dissociation in buffer without peptide. The *K_D_* value of HGP-1 was 1.73 × 10^−6^ M (697.5 1/Ms for *k_a_* and 0.001243 1/s for *k_b_*).

The binding specificity of HGP-1 was investigated by a fluorescence-based ELISA assay. HGF was coated on the plate as the solid phase, and 10 μM FITC-labeled HGP-1 coupling with different concentrations of cytokines (EGF, VEGF, bFGF) and BSA acted as liquid phase. The proteins except HGF did not obviously disrupt the binding of HGP-1 to immobilized HGF (Figure 3b). Although HGP-1 displayed on bacteria surface showed a high binding activity with EGF (Supplementary Figure S2b), the data from fluorescence-based direct ELISA gave an opposite result. Even at a high concentration (100 μM), HGP-1 did not exhibited a binding level to EGF as high as to HGF. The RFU readouts of the wells coated with EGF were approximately 8 times lower than the ones with HGF post HGP-1 incubation (Figure 3c). Furthermore, MTT assay was used for the detection of HGP-1 influence on EGF-dependent cell proliferation to further evaluate the binding capability of HGP-1 to EGF. In this assay, A549 cells were used, on which EGFR is over-expressed. The MTT results illustrated that HGP-1 performed no significant inhibition on the EGF-dependent cell proliferation (Supplementary Figure S4), which indicated that HGP-1 might not bind to EGF or at least not bind to the receptor-binding site of EGF.

### HGF targeting peptides inhibited HGF-dependent cell proliferation

**Figure 4:**
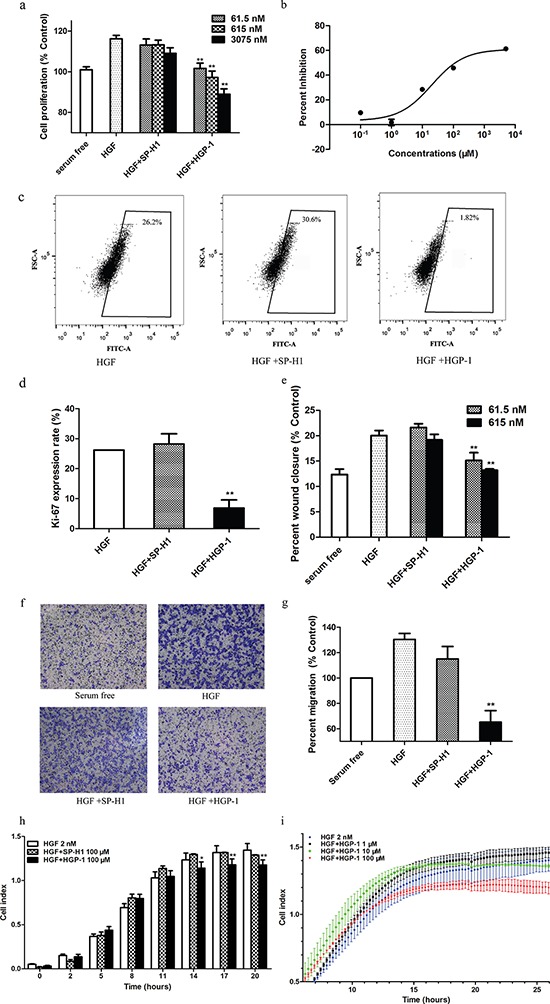
HGP-1 attenuated HGF-mediated cellular functions **a.** Measurement of cell proliferation treated by HGP-1 coupled with HGF (0.625 nM) in A549 cells for 4 days by MTT assay (*n* = 3). **b.** Inhibition on HGF (1 nM)-mediated proliferation by HGP-1 (0.1 μM to 5000 μM) post 4-day treatmentx examined by MTT assay (*n* = 4). **c.** Representatives of Ki-67 expression levels of A549 cells evaluated by flow cytometry after HGP-1 (1 μM) treatment. **d.** Quantitation of Ki-67 expression in A549 cells post HGP-1 (1 μM) 2-day treatment (*n* = 3). **e.** Quantitation of MDA-MB-435s cells migration post HGP-1 (1 μM) 2-day treatment determined by wound healing assay (*n* = 5). **f.** Representative photographs of transwell assay. **g.** Quantitation of MDA-MB-435s cell migration rate post HGP-1 (1 μM) 18-hour treatment evaluated by transwell assay (*n* = 3). **h.** Determination of A549 cells migration treated by HGP-1 (100 μM) in RTCA (*n* = 3). **i.** Measurement of A549 HGF-initiated cells motility post HGP-1 treatment in concentration ranges by RTCA (*n* = 3). SP-H1 group in each analysis worked as control. Values were expressed as mean ± SEM. *P < 0.05, ** 0.01 < *P* < 0.05, represented the significance between HGF and SP-H1 or HGP-1 groups.

The HGF/MET axis has been implicated in cell proliferation [3]. Thus, we would like to assess the HGP-1 inhibition on cell proliferation initiated by HGF via MTT assay and Ki-67 expression evaluation. After 4 days of HGP-1 treatment, the results from MTT assay demonstrated that A549 cells treated with HGP-1 had 10% to 25% reduction in proliferation at the range of HGP-1 concentrations (61.5 nM to 3.075 μM) as shown in Figure 4a. It was further validated that HGP-1 could inhibit A549 cells proliferation with an IC_50_ value of 496.4 μM (Figure 4b). As a supplement, another HGF targeting peptide HGP-2 had a similar inhibitory effect on cell proliferation in A549 cells although its binding capability to HGF was lower than HGP-1 (Supplementary Figure S5). Moreover, HGP-1 and HGP-2 did not exhibit detectable cytotoxicity to cell proliferation in HGF-free environment (Supplementary Figure S6a and S6b).

Ki-67 is a nucleoprotein whose expression is associated with cell proliferation. Ki-67 expression levels in A549 cells after treated with peptides were determined by flow cytometry. The results illustrated that Ki-67 expression of A549 cells had a 74% decline when treated with 1 μM HGP-1 for 2 days. In contrast, 1 μM SP-H1 did not show obvious inhibitory effect on Ki-67 expression compared with HGP-1 group (Figure 4c and 4d).

### Cell migration was inhibited by HGF targeting peptides

HGF is a potent factor that promotes cell migration and invasiveness [22]. To assess the inhibitory effect of HGP-1 on migration, wound healing assay, transwell assay and real-time cellular analysis were applied. In wound healing assay and transwell assay, MDA-MB-435s cells were used referring to the previous description [23, 24]. The results of wound healing assay showed that HGP-1 performed a potent inhibitory effect on MDA-MB-435s cell migration, and the inhibition displayed decrease trend with treatment by increasing concentration of peptides (Figure 4e and Supplementary Figure S7a). HGP-2 also displayed a similar effect on MDA-MB-435s (Supplementary Figure S7a and Supplementary Figure S7b). Besides, no obvious inhibitory effect of HGP-1 and HGP-2 on MDA-MB-435s migration was detected in the culture system without HGF (Supplementary Figure S8).

The cell movement from one side of transwell membrane to the basal side could mimic cell vertical motility. To further validate the migration inhibitory effect of HGP-1 on MDA-MB-435s, a transwell migration assay was conducted. As shown in Figure 4f and 4g, after 18-hour treatment, the number of migrated cells in HGP-1 group had almost 50% reduction comparing with cells treated by HGF alone.

Label-free real-time cell-based assay (RTCA) could provide cell dynamic data. Therefore, it was used to examine the time-dependent effect of HGP-1 on A549 cell motility [25]. A549 cells were treated by HGF alone or coupling with 100 μM peptide (HGP-1 or SP-H1) to assess temporal inhibitory effect of HGP-1 on cell migration. When compared with SP-H1 treatment, 100 μM HGP-1 reduced the cell index by 40% at the 20^th^ hour (Figure 4h), which meant that HGP-1 could inhibit the migration of A549 cells induced by HGF effectively. In addition, we noticed that inhibitory effect of HGP-1 on cell migration started approximately 14 hours post treatment.

Furthermore, RTCA was used to evaluate whether inhibitory effect of HGP-1 on cell migration was concentration-dependent. The result demonstrated that HGP-1 at the concentration of 10 μM and 100 μM established inhibitory effect on cell migration (Figure 4i) in a concentration-dependent manner. In RTCA, HGP-1 exhibited a temporal and concentration-dependent inhibitory effect on A549 cell migration.

### HGP-1 inhibited tumor growth in luciferase-expressed A549 xenograft models

**Figure 5:**
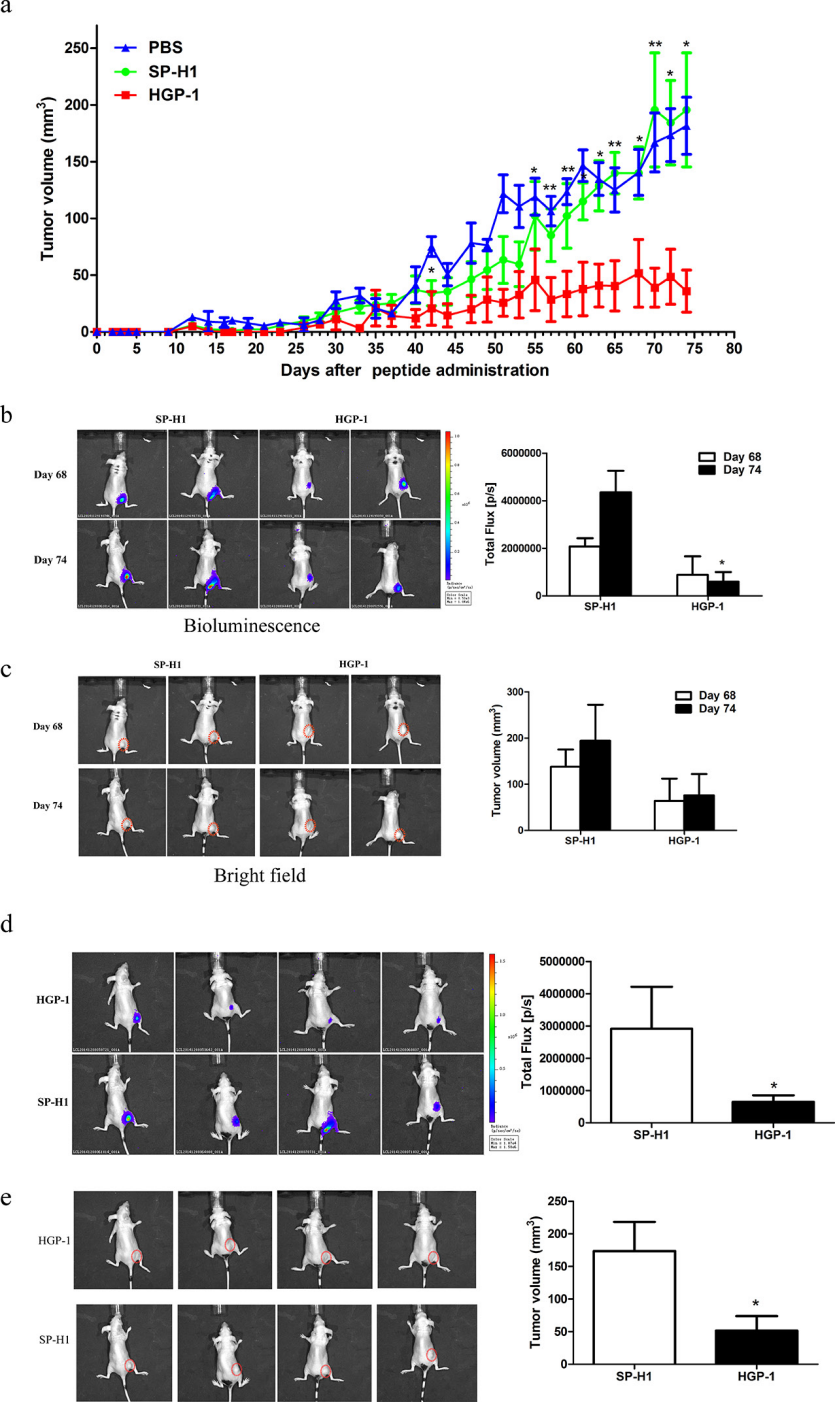
HGP-1 inhibited tumor growth *in vivo* Peptides were subcutaneously injected in dosage of 10 mg/kg every other day for 6 times 2 days post tumor transplantation. **a.** Data of tumor volumes post peptides administration for 74 days. **b.** Representatives bioluminescence images of tumor-bearing mice in two different time points. Quantitation of tumor volumes was determined by total flux. **c.** Representatives bright images of tumor-bearing mice in two different time points. Quantitation of tumor volumes was performed by vernier calipers. **d.** Representatives bioluminescence images of tumor-bearing mice on day 74 post peptide administration. Quantitation of tumor volumes was assessed by total flux. **e.** Representatives bright images of tumor-bearing mice on day 74 post peptide administration. Quantitation of tumor volumes was determined by vernier calipers. The circle in red presented the location of tumor (The size of the red circle was not associated with tumor volume). Values were mean ± SEM (*n* = 8 mice per group). *P < 0.05, **0.01 < *P* < 0.05, represented the significance between SP-H1 and HGP-1 group in the same day.

In order to assess the *in vivo* activities of HGP-1, luciferase-expressed A549 subcutaneous tumor xenograft model was established. The tumor-bearing mice were subcutaneously administrated with 10 mg/kg HGP-1 or SP-H1 once every other day for 6 times two days post transplantation. Results showed that the early HGP-1 treatment would postpone the initiation of tumors and retard the tumor growth (Figure 5a). Data from the ROI analysis of tumor bioluminescence signals displayed the same trend with those from vernier calipers on the assessment of tumor volume alteration (Figure 5b, 5c). The average volume of tumor in day 74 of HGP-1 group exhibited a 77% reduction compared with SP-H1 group in the bioluminescence analysis, in accordance with data from vernier calipers (Figure 5d, 5e). No significant inhibitory effect of HGP-1 on tumor metastasis was observed (Data not shown).

### Inhibitory effect of HGP-1 on cell activities was via affecting MET signaling

**Figure 6:**
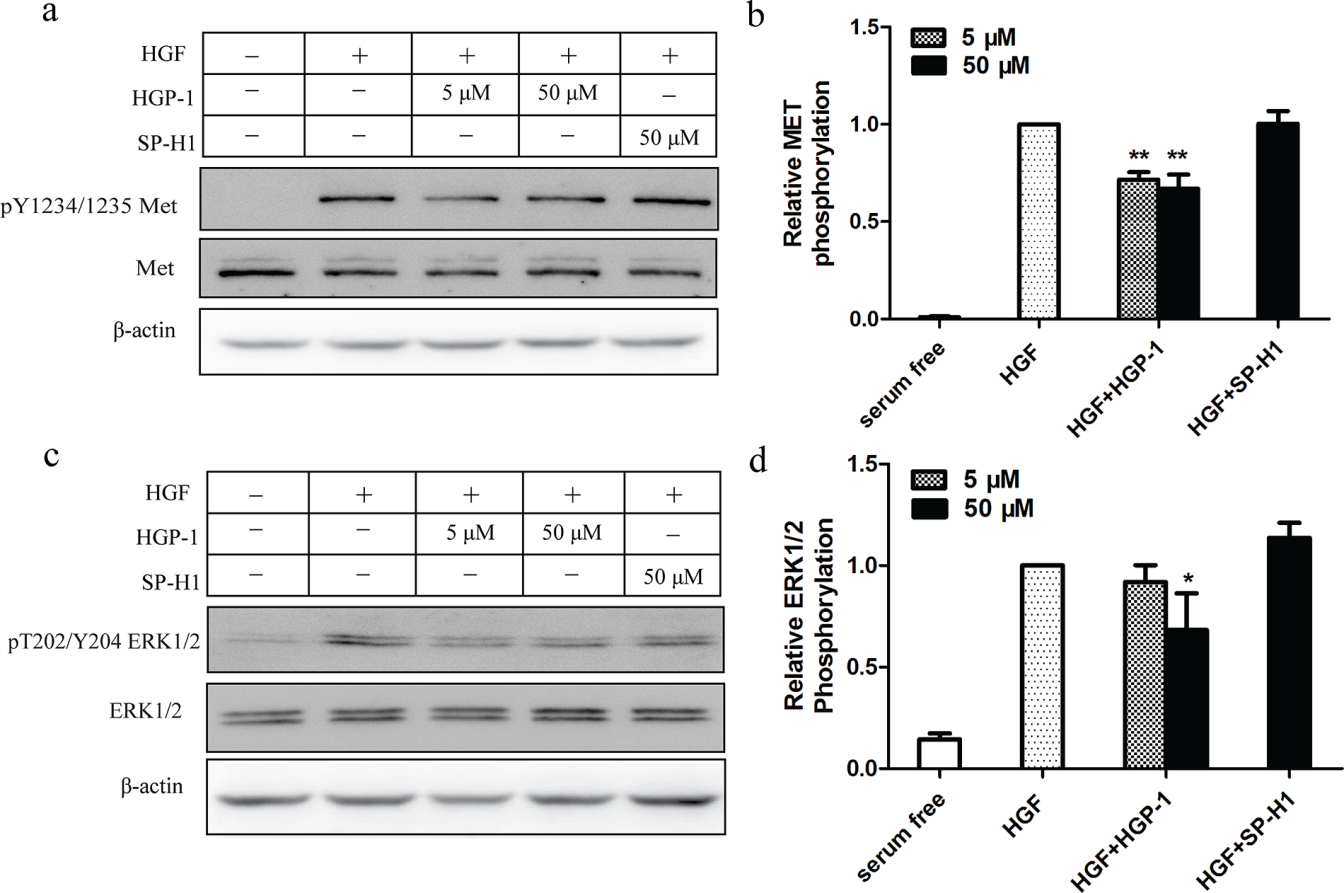
HGP-1 attenuated HGF-mediated phosphorylation of MET and downstream signaling The cells were treated with HGP-1 (5 μM and 50 μM) and SP-H1 (50 μM) for 15 minutes respectively. **a.** Representatives Western Blot of phospho-MET level in A549 cells after treated by HGF alone or combining with HGP-1. **b.** Quantitation of HGP-1 inhbitory effect on phospho-MET level in A549 cells. **c.** Representatives Western blot of phospho-ERK1/2 level in A549 cells after HGP-1 treatment. **d.** Quantitaion of HGP-1 inhibitory effect on phospho-ERK1/2 level in A549 cells. *P < 0.05, **0.01 < *P* < 0.05, represented the significance between HGF and HGP-1 or SP-H1 group. Values were mean ± SEM (*n* = 5).

It is well documented that HGF/MET signaling cascade is associated with cell proliferation and motility [2]. To explore how HGP-1 inhibited cell proliferation and migration through HGF/MET signaling, A549 cells pre-treated by HGP-1 were lysed, and their phosphorylation level of MET and ERK1/2 were measured by Western Blot. The result showed that the phospho-MET level at Tyr1234/1235 would decrease by 28% and 33% after treated by 5 μM and 50 μM HGP-1 (Figure 6a and 6b), while phospho-ERK1/2 at Thr202/Tyr204 was reduced by 13% and 31% (Figure 6c, 6d). HGP-1 could inhibit the phospho-MET level at the concentration of 5 μM and display significant inhibitory effect on phosphorylation of ERK1/2 at the concentration of 50 μM without affecting expression of MET and ERK1/2 (Supplementary Figure S9a). In sum, the inhibitory effect of HGP-1 on cell proliferation and migration stimulated by HGF was via affecting the activity of MET signaling. The result from structure simulation through ZDOCK illustrated that HGP-1 might bind to the C-terminal of HGF-β (Supplementary Figure S10), which was close to the binding site of HGF-β to MET [26]. It provided a possible explanation for the inhibitory effect of HGP-1 on the activation of HGF/MET signaling.

## DISCUSSIONS

HGF/MET axis regulates cell proliferation, migration and morphogenesis [3, 27]. The dysregulation of this pathway has been implicated in cancer initiation, development and even metastasis [28], which suggested that targeting to HGF/MET axis is a potential strategy in cancer targeted therapy. In this article, we obtained a series of HGF targeting peptides and identified a peptide candidate named HGP-1. HGP-1 either on the surface of bacteria clone or synthesized, exhibited a moderate affinity and good specificity for binding to HGF. As a HGF binder, HGP-1 could inhibit cell proliferation both *in vitro* and *in vivo* and migration at least *in vitro* via reducing the phosphorylation level of MET and ERK1/2.

In 2009, Eric M. and colleagues reported a HGF-β targeting peptide that could work as a noncompetitive inhibitor for MET signaling [24]. In our study, we also presented a peptide candidate HGP-1 for HGF/MET signaling inhibition. HGP-1 could target to the full length HGF with *K_D_* of 1.73 μM, lower than the disulfide-constrained HGF-β targeting peptide of HB1 (3 μM) in the first panning. In addition, although HGF-β associated with the activation of MET, but the activation of MET need a cooperative interaction between α- and β-chain of HGF [29, 30]. That is the reason why we choose the full length HGF to be the targeting protein in our screening.

In order to study the mechanisms of inhibitory effect of HGP-1 *in vitro* and *in vivo*, phosphorylation level of MET and its downstream components were detected. Results from Western Blot illustrated that HGP-1 could inhibit phosphorylation of MET at Y1234/1235, which provided a possible explanation for alteration of cell activities induced by HGP-1. According to the report from Roskoski and Trusolino et al., phospho-MET would lead to the activation of Ras-ERK cascade in HGF/MET signaling and ERK1/2 involved in the regulation of cell proliferation [27, 31]. In our study, HGP-1 inhibited the phospho-ERK1/2 level at T202/Y204, which indicated that ERK1/2 may be one of the mediators for HGP-1 to affect cell proliferation. As an important downstream protein, ERK1/2 could activate myosin light chain kinase to manipulate cell motility [32], which indicated that ERK1/2 could be involved in HGP-1 inhibitory effect on cell migration initiated by HGF. Belonging to ERK family, ERK2 performed a dominant role in governing cell proliferation and migration [33]. In this study, 50 μM HGP-1 inhibited phospho-ERK2 level at a similar extent to 5 μM HGP-1 (Supplementary Figure S9b), which meant that increased HGP-1 concentration was not a main factor that cause the drop of phospho-ERK2 level. The inhibition of HGP-1 on phospho-ERK2 might be a possible explanation for the decline of A549 migration when treated by 10 μM HGP-1 in RTCA. However, some other molecules might be involved in the decrease of A549 migration induced by 100 μM HGP-1, because 50 μM HGP-1 did not induce more significant down-regulation of phospho-ERK2 level compared with 5 μM HGP-1. It could be explained that there may exist other downstream signaling components in HGF/MET signaling and other possible receptors or lateral signaling pathways such as EGFR pathway [34] involved in the inhibitory effect of HGP-1.

In our study, ROI analysis of bioluminescence signal and vernier calipers measurement were applied to measure tumor volumes. The data from bioluminescence provided more objective assessment on tumor volumes, which would be a better approach to observe the tumor growth. The bioluminescence signal did not provide evidence for HGP-1 inhibitory effect on metastasis. It might indicate that HGP-1 did not have apparent inhibitory effect on tumor metastasis at least with the concentration applied. On the other hand, considering the small volume of metastasis, the emitted bioluminescence signal might not be observed by IVIS Lumina II system. Although no obvious metastasis signal was recorded via bioluminescence, we noticed that some phenomena occurred which might be a consequence of metastasis. Two mice in SP-H1 group presented limb movement disorder in half side, along with wry neck, circle motion and rapid weight loss on day 43 and day 47, but no mice in HGP-1 group appeared similar phenomena. It has been reported that brain metastases from cervical carcinoma would lead to Hemiballismus [35]. Therefore, we speculated that the limb movement disorder of the two mice from SP-H1 might be the result from brain metastasis.

Taken together, we identified a peptide targeting to HGF, which could behave as an inhibitor to HGF/MET signaling. The HGF targeting peptide candidate HGP-1 could inhibit cell proliferation and migration via down-regulating the phosphosrylation levels of MET and ERK1/2. Our studies revealed that peptides might be effective targeting molecules for cancer targeted therapeutics.

## MATERIALS AND METHODS

### Reagents and cell culture

Recombinant human full length HGF (10463- NHAC-A), VEGF-165 (11066-HNAB), Protein G (13103-PNAE), Biotinylated recombinant human full length HGF (10463-HNAC-B), HGF antibody (10463-RP01-B), MET antibody (10692-MM02), EGF antibody (0605-R008) were purchased from Sino Biological Inc. Recombinant human EGF (H6000–10), bFGF (H3000–10) were obtained from Beijing Wishbiotechnology Co., Ltd. Dynabeads MyOne streptavidin C1 magnetic beads (#65002) and Streptavidin Phycoerythrin Conjugates (S-866) were obtained from Invitrogen. Magnet (MS-12) was purchased from Shanghai Allrun Nano Science & Technology Co., Ltd. Human serum (ZX101–1) was bought from Beijing Zoman Biotechnology Co., Ltd. RPMI-1640 medium (SN30809.01B), high glucose Dulbecco's modified Eagle's medium (DMEM, SN30022.01B), penicillin/streptomycin solution (SV30082.01) and fetal bovine serum (FBS, SV30087.02) were purchased from Hyclone. Collagen type I from rat tail (C7661–5MG) was bought from Sigma-Aldrich. Bovine serum albumin V (BSA, A8020) was obtained from Solarbio. Ki67 cell proliferation Detection Kit (KGA325) was purchased from KeyGENE BioTECH. NP-40 lysis buffer (P0013F), phosphorylated p44/42 MAPK antibody (AM071) and p44/42 MAPK antibody (AM076) were obtained from Beyotime Institute of Biotechnology. Phosphorylated MET antibody (#3129) was purchased from Cell Signaling Technology. Amino coupling reagent kit (2060499) was purchased from GE healthcare.

Human non-small lung cancer cell line A549 (TCHu150, Chinese Academy of Sciences Cell Bank) was maintained in RPMI-1640 medium. Human melanoma cell line MDA-MB-435s (TCHu 36, Chinese Academy of Sciences Cell Bank) was cultured in high glucose DMEM. The medium were supplemented with 10% FBS and 1% penicillin/streptomycin. All the cells were cultured at 37°C, in 5% CO_2_.

### Screening of HGF binding peptide from a random library

The bacteria display library of linear peptides was a gift from Professor Patrick S. Daugherty (University of California, Santa Barbara, United States). In this library, peptide with 15 randomized amino acid positions (X15) was displayed at the N-terminus of eCPX [36] as fusion protein on the surface of *Escherichia coli*. The size of library was 5 × 10^8^.

The frozen stock was thawed and cultivated overnight at 37°C in Luria-Bertani medium (LB medium) supplemented 34 μg/mL chloramphenicol and 0.2% (w/v) D-(+)-glucose with shaking at 200 rpm. The bacteria were subcultured 1:50 the next day for 2 hours in LB medium at 37°C with shaking at 200 rpm, and then induced by 0.02% (w/v) L-(+)-arabinose for 1 hour at room temperature to express the outer membrane protein scaffold. The MACS was conducted with 42.5 nM biotinylated HGF to reduce the size of library. When the size of library was smaller than 10^7^, FACS was performed. The enriched library incubated with biotinylated HGF at concentrations ranging from 20 nM to 5 nM to enrich the high affinity HGF binding peptides at 4°C for 45 minutes. Subsequently, Streptavidin R-phycoerythrin (SAPE) at a final concentration of 3.3 nM was added to the mixture following by the incubation at 4°C for 30 minutes. The PE-A fluorescence intensity of each sample was measured by FACS Aria II (Becton Dickinson). The bacteria with high PE-A fluorescence intensity were sorted and cultured in LB medium overnight for another cycle of sorting. In the last several cycles of sorting, 10% human serum was supplemented to increase the sorting stringency [37]. The bacteria sorted from the last cycle of sorting were plated on a LB-agar plate for an overnight incubation. Bacteria clones were obtained and sent to GENEWIZ Inc. for DNA sequencing.

### Binding capability and specificity analysis of peptides displayed on the bacteria surface

Bacteria clones were subcultured and induced as described before. Then, 10^7^ bacteria of each clone were incubated with biotinylated HGF referring to sorting procedure. After incubating with HGF, bacteria were divided into two groups. The ‘original’ group was washed referring to sorting procedure, and the ‘washed’ group would have a tough washing. After centrifuged at 3000 × g for 5 minutes, bacteria in ‘washed’ group were resuspended by 1 mL phosphate buffered saline (PBS, PH 7.4) and inverted at 4°C for 10 minutes. The washing steps were repeated for 3 times. Samples from two groups were analyzed by flow cytometry.

To examine the binding specificity of peptide on the bacteria surface, 10^7^ bacteria were incubated with 7 nM HGF alone or coupled with the same molarity of VEGF-165, EGF, bFGF or BSA separately. The washing steps were performed following sorting procedure. The fluorescence signals of samples were recorded with flow cytometer.

### Examination of binding property of soluble peptide

Peptides were synthesized in Shanghai Bootech Bioscience & Technology Co., Ltd., and FITC was labeled at the N- terminal of the peptide sequence. The binding property of soluble peptides was determined by fluorescence-based ELISA. Plates (#3925, Corning) were coated with 15.6 nM of HGF or EGF in PBS (pH 7.4) overnight at 4°C. Then, wells were blocked with 5% (m/v) BSA for 1 hour with shaking at room temperature. Different concentrations of FITC-labeled peptide (0.1 μM, 1 μM, 10 μM and 100 μM) were added to the wells respectively for 1.5 hours incubation at room temperature. At last, the fluorescence intensity of each well was determined by VECTOR X4 Multilabel Plate Reader (PerkinElmer).

To evaluate the specificity of soluble peptide, a fluorescence-based ELISA was established. HGF was coated on the plate with concentration of 14.3 nM in PBS (pH 7.4) overnight at 4°C. Afterwards, plates were blocked with 5% BSA for 1 hour with shaking at room temperature. FITC-labeled peptide (10 μM) coupling with different concentrations (0.05 nM, 0.5 nM, 5 nM and 50 nM) of HGF, VEGF-165, EGF, bFGF or BSA was added into wells separately and incubated at room temperature for 1.5 hours. The fluorescence intensity of each well was determined by VECTOR X4 Multilabel Plate Reader (PerkinElmer).

### Measurement of *K*_D_ for peptide binding to HGF

Kinetic (*k_a_* and *k_d_*) and affinity constant (*K_D_*) of soluble peptide were determined by surface plasmon resonance technique on a Biacore X100 instrument (GE healthcare). The research-grade CM5 sensor chip was activated following the process which was recommended in the amino coupling reagent kit. Human full length HGF was dissolved in PBS and diluted to a suitable concentration by 20 mM NaCOOH buffer (pH4.0) and immobilized on the chip, and 2000 resonance units were immobilized. The remaining reactive groups were inactivated by ethanolamine. The peptide was dissolved and diluted by HBS-EP (100 mM HEPES, 150 mM NaCl, 3 mM EDTA-Na_2_ and 0.005% P20, pH7.4). Samples composed of concentrations ranging from 0 to 100 μM of peptides were injected over the protein surface. Each sample was injected at a flow rate of 30 μL/min for 2 minutes and followed by 8 minutes of dissociation in HBS-EP buffer without peptide [38]. *K_a_*, *K_d_* and *K_D_* were calculated by Biacore X100 Evaluation software.

### MTT assay

A549 cells were seeded in a 96-well plate at a density of 7000 cells per well. After overnight culture, cells were starved with serum free DMEM medium for 48 hours. HGF (0.625 nM) alone or mixed with concentrations ranging from 61.5 nM to 3.075 μM of peptides was added into the cells post starvation. After cultured for 4 days, the cell culture medium was changed with fresh medium containing 0.5 mg/mL 3-(4, 5-Dimethylthiazol-2-yl)-2, 5-diphenyltetrazolium bromide (MTT) solution for another 4 hours incubation at 37°C, in 5% CO_2_. Then, medium was removed and cells were lysed with dimethyl sulfoxide [39]. The absorption of the solution in each well was measured at 490 nm on a VECTOR X4 Multilabel Plate Reader. IC_50_ value was also determined and calculated by GraphPad Prism 5.

MTT assay was also used to assess the cytotoxicity of peptides after 4-day incubation with peptides at different concentrations in the medium with 10% FBS. The cells were seeded in the 96-well plate at density of 2000 cells per well.

### Measurement of Ki-67 expression levels by flow cytometry

A549 cells were cultured on a 12-well plate (2 × 10^5^ cells/well), grown to a 70–80% confluent cell monolayer, and serum-starved for 24 hours. Fresh medium containing 1 nM HGF alone or mixed with 1 μM peptides was added into each well for 2 days treatment. Afterwards cells were detached by 0.1% trypsin and fixed by ethanol for 2 hours at 4°C. Then, cells were incubated with Ki-67 antibody following the instruction of the Ki67 cell proliferation Detection Kit. The FITC fluorescence intensity of each sample was determined by flow cytometry.

### Assessment of cell migration by scratch wound assay

MDA-MB-435s cells were plated on a 24-well plate (1 × 10^5^ cells/well), grown to an 80–90% confluent cell monolayer, and serum-starved for 24 hours. Wounds were scratched gently with tip of a plastic pipet and washed twice with PBS. Subsequently, HGF (0.625 nM) alone or coupled with peptides at the concentrations of 61.5 nM and 615 nM were added into the wells for 2 days treatment. Each well was photographed at 10 × magnification with light microscope before and after peptide treatment. The cell migration capabilities were assessed by calculating the width alteration of the wound area by Photoshop [25]. The percentage of cell migration followed the equation:

$$\text{Percent migration}=\frac{\begin{array}{c} \text{width before treatment}-\\ \text{width after treatment} \end{array}}{\text{width before treatment}}\times 100\%$$

Wound healing assay was applied to evaluate the inhibitory effect of peptides to cell migration after one-day incubation with peptides at different concentrations in medium with 10% FBS.

### Assessment of cell migration by transwell assay

MDA-MB-435s cells were detached by 0.25% trypsin and diluted with serum-free medium to a final concentration of 4 × 10^5^ cells/mL post 24 hours serum starvation. Serum free medium containing 1 nM HGF alone or mixed with 1 μM peptides was added to wells in 24-well plate (bottom chamber). The basal side of membrane in the millicell chamber (PIEP12R48, Millipore) was pre-coated with 10 μg/mL rat tail collagen type I, and 0.1 mL cell suspension was added to millicell. The millicell chambers were hanged over the wells and cultured at 37°C, 5% CO_2_. After cultured for 18 hours, cells on the basal side of the membrane were fixed, stained with 0.5% (w/v) crystal violet and photographed. The crystal violet in cells were then eluted and quantified by measuring absorbance at 595 nm on the VECTOR X4 Multilabel Plate Reader [24, 26].

### Assessment of cell migration by real-time cellular analysis

A549 cells were detached and diluted with serum-free medium to a final concentration of 8 × 10^5^ cells/mL post 24 hours serum starvation. The upper chamber of CIM-16 plate was pre-coated with 10 μg/mL rat tail collagen type I. Serum-free medium containing 2 nM HGF alone or with peptides at concentrations ranging from 1 μM to 100 μM was added into the bottom chamber of CIM-plate. Afterwards, the device including upper chamber hanging on the lower chamber was balanced at 37°C, in 5% CO_2_ for 1 hour and baseline was measured. Before data recording, cell suspension was added into the upper chamber and incubated at room temperature for 30 minutes. Cell index was measured every 15 minutes during 22 hours on the xCELLigence DP system (ACEA Biosciences, Inc.) [40].

### Assessment of tumor growth in mice xenograft model

Four to five-week-old female Balb/c nu mice (SLAC Laboratory Animal) were used in the experiment. Mice were injected subcutaneously in the flank with 2 × 10^6^ luciferase-expressed A549 cells in 0.1 mL PBS. When the tumor volumes reached 200 mm^3^, the mice were sacrificed. Tumor mass was separated under aseptic conditions and washed by PBS. After removing the thanatosis tissue, tumor tissues were cut into small cubes (approximately 1 mm^3^) and transplanted to mice right hindpaw [41]. The mice were randomized into SP-H1 (Scramble peptide of HGP-1) and HGP-1 group (8 mice per group). Peptides were subcutaneously administrated (10 mg/kg) every other day for 6 times two days post transplantation. The tumor volumes and the weights of mice were measured every 2–3 days. Tumor volume = 1/2 × a × b^2^ (a is the length, b is the width). The mice were photographed in an IVIS Lumina II system (Caliper LifeScience) and ROIs of bioluminescence were analyzed to evaluate tumor growth at two different time points. All animal studies were approved by a local Ethics Committee for Animal Experiments.

### Detection of phosphorylation of MET and ERK1/2 by Western Blot

A549 cells were cultured in a 6-well plate (2 × 10^5^ cells/well), grown to an 80–90% cell confluent and serum-starved for 24 hours. Medium containing 2 nM HGF alone or coupled with HGP-1 at concentrations of 5 μM and 50 μM were added to wells for 15 minutes treatment. Afterwards, cells were lysed with NP-40 lysis buffer (supplemented with 1 nM PMSF). The Western Blots were conducted as described [23] with the following antibodies: phosphorylated MET, MET, phosphorylated p44/42 MAPK, p44/42 MAPK.

### Structure prediction and model construction of HGP-1

Three-dimensional models of the HGP-1 were constructed through the Local Meta-Threading Server (http://zhanglab.ccmb.med.umich.edu/LOMETS/) [42] for the selection of the best model with the highest confidence score. The structure of the complex between the HGP-1 and HGF-β was predicted and described by ZDOCK, a protein-docking algorithm (http://zlab.umassmed.edu/zdock/) [43].

### Statistics analysis

All the data were expressed as means ± SEM. Histogram and line charts were generated by GraphPad Prism 5. *T*-test and ANOVA were used to determine the *P* values. *P*-value < 0.05 was considered to be statistically significant.

## SUPPLEMENTARY MATERIALS FIGURES


