# Supplementary material for: The HGF inhibitory peptide HGP-1 displays promising in vitro and in vivo efficacy for targeted cancer therapy
Source: Oncotarget. 2015 May 11;6(30):30088–101. doi: 10.18632/oncotarget.3937 (PMC4745783; doi:10.18632/oncotarget.3937)
Supplement: Supplementary file 1 [file oncotarget-06-30088-s001.pdf]

SUPPLEMENTARY FIGURES

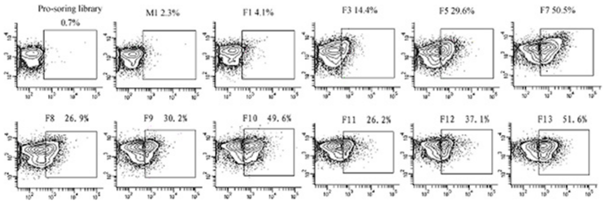

Supplementary Figure S1: Enrichment procedure of HGF binding peptides in 13 cycles of sorting by FACS.

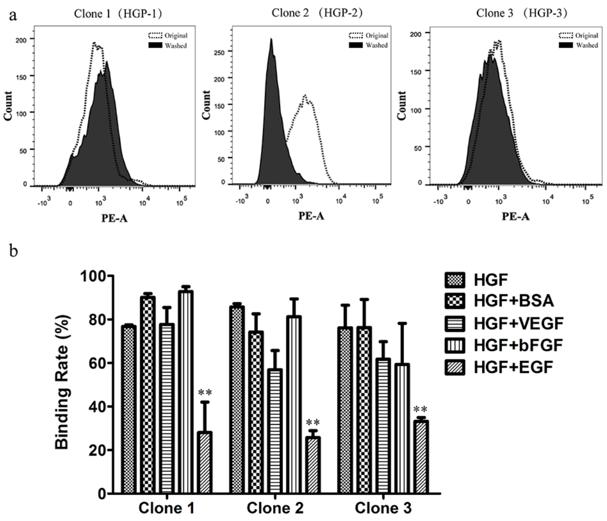

Supplementary Figure S2: Binding characteristics of peptides displayed on the surface of bacteria to HGF. a. Peptide binding capability to HGF on the surface of bacteria was determined by flow cytometry. Bacteria in the "original" group represented the bacteria had a regular washing procedure and that in "washed" group had a tough washing procedure. **b.** Binding specificity of peptides to HGF on the surface of bacteria examined by flow cytometry. (*n* = 3).

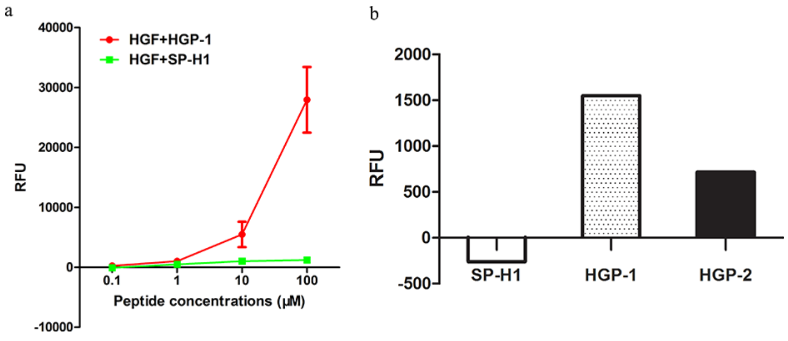

Supplementary Figure S3: Binding properties of peptide to HGF determined by fluorescence-based ELISA assay. a. Binding capability of SP-H1 to HGF at the concentrations of 0.1 μM, 1 μM, 10 μM and 100 μM measured by ELISA (*n* = 3). **b.** Binding capability of soluble peptides determined by ELISA. Values were mean ± SEM.

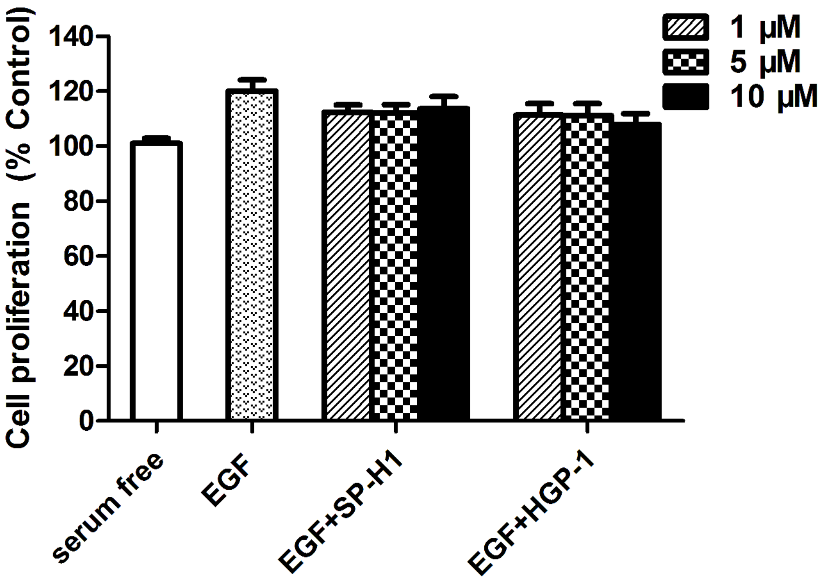

Supplementary Figure S4: HGP-1 (1 μM, 5 μM and 10 μM) exhibited no inhibitory effect on A549 cell proliferation initiated by EGF (20 nM) post 3-day treatment in MTT assay. Values were mean ± SEM.

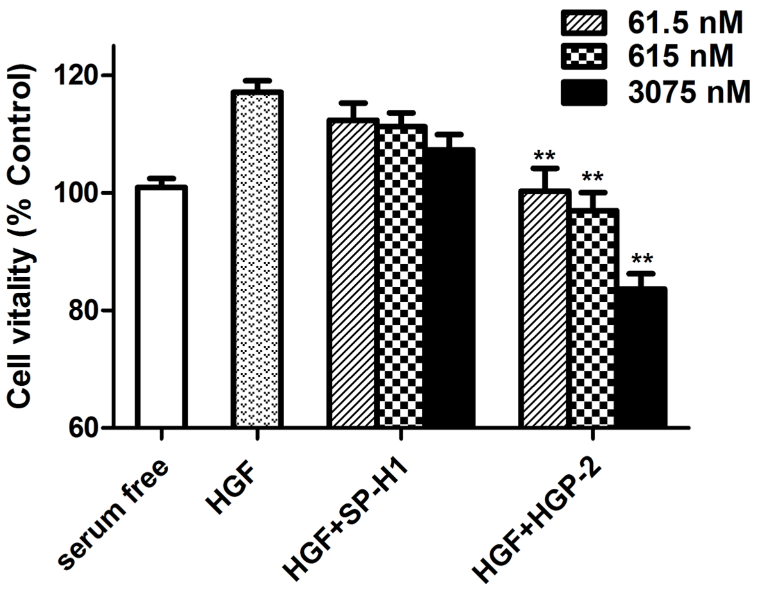

Supplementary Figure S5: HGP-2 (61.5 nM, 615 nM and 3.075 μM) inhibited A549 cells proliferation in 4-day treatment measured by MTT assay (n = 3). ** $0.01 < P < 0.05$, represented the significance between HGF and SP-H1 or HGP-2 groups. Values were mean ± SEM.

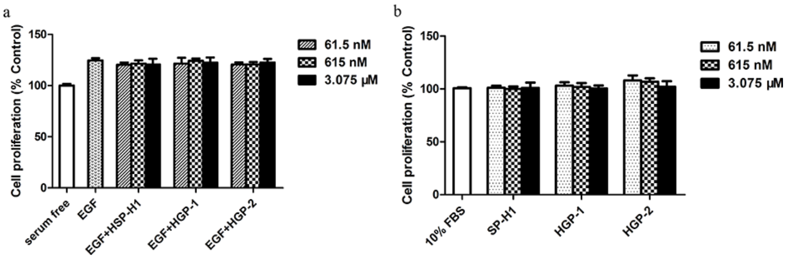

Supplementary Figure S6: Evalutation of peptide cytotoxicity. a. Evaluation of inhibitory effect of peptides (61.5 nM, 615 nM and 3.075 μM) on A549 cell proliferation induced by EGF in MTT assay post 3-day treatment (n = 4). **b.** Assessment of peptides (61.5 nM, 615 nM and 3.075 μM) cytotoxicity to A549 cell proliferation post 4-day treatment by MTT assay (n = 4). Values were mean ± SEM.

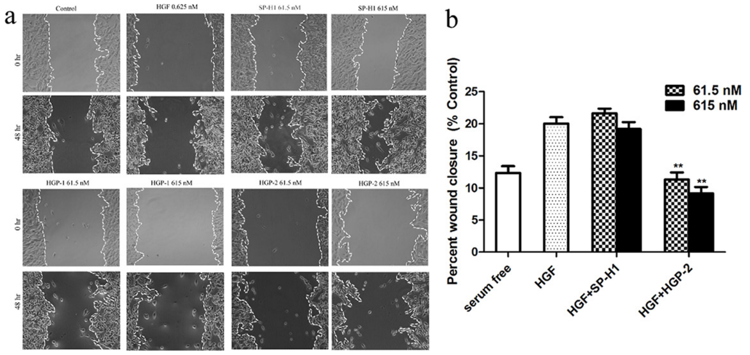

Supplementary Figure S7: HGP-2 inhibited MDA-MB-435s migration measured by wound healing assay. a. Representatives photographs (10 ×) of cell migration in MDA-MB-435s determined by wound healing assay. **b.** Quantitation of cell migration in MDA-MB-435s post HGP-2 treatment determined by wound healing assay (n = 3). ** 0.01 < P < 0.05, represented the significance between HGF and peptide groups. All values were expressed as the mean ± SEM.

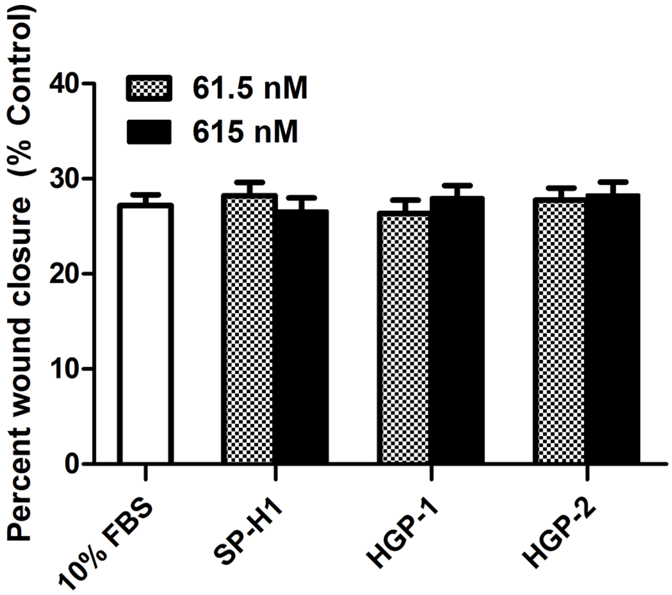

Supplementary Figure S8: Evaluation of non-specific inhibition of peptides (61.5 nM and 615 nM) on MDA-MB-435s migration by wound healing assay post one day treatment (n = 3). Values were mean ± SEM.

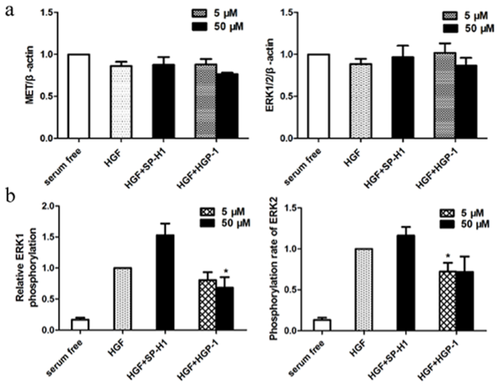

Supplementary Figure S9: Quantitation of the results from western Blots. a. Quantitation of expression level of MET and ERK1/2 post HGP-1 treatments in Western Blot. **b.** Quantitation of phospho-ERK1 and phospho-ERK2 level post HGP-1 treatment in Western Blot. * $P < 0.05$, represented the significance between HGF and peptide groups. All values were mean ± SEM.

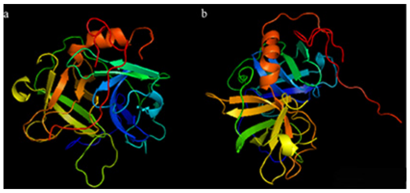

Supplementary Figure S10: Simulation of HGP-1 binding to HGF β in ZDOCK. String in red presented HGP-1. **a.** front view. **b.** side view.
